# Impact of cancer in patients with aortic stenosis undergoing transcatheter aortic valve replacement: A systematic review and meta-analysis

**DOI:** 10.1016/j.ijcha.2024.101410

**Published:** 2024-04-16

**Authors:** Takumi Osawa, Kazuko Tajiri, Tomoya Hoshi, Masaki Ieda, Tomoko Ishizu

**Affiliations:** a Department of Cardiology, Institute of Medicine, University of Tsukuba, Japan; b Department of Cardiology, Tsukuba Medical Center Hospital, Japan; c Department of Cardiology, National Cancer Center Hospital East, Japan; d Tsukuba Life Science Innovation Program (T-LSI), School of Integrative and Global Majors (SIGMA), University of Tsukuba, Japan; e Department of Cardiology, Keio University School of Medicine, Japan

**Keywords:** Cardio-oncology, TAVI, TAVR, Onco-cardiology

## Abstract

**Background:**

Owing to the minimally invasive nature of transcatheter aortic valve replacement (TAVR), TAVR seems to be preferred in patients with cancer; however, related research on the clinical efficacy and safety of TAVR in patients with cancer and severe aortic stenosis is limited, and conclusions are controversial. This study aimed to evaluate the clinical outcomes of patients with cancer who underwent TAVR.

**Method and results:**

We conducted a systematic review and meta-analysis to investigate the clinical outcomes in patients with and without cancer who underwent TAVR. We systematically reviewed and analyzed 15 studies (195,658 patients) published in PubMed and Cochrane Library databases between January 2022 and January 2023. The primary outcomes were short-term (in-hospital or 30-day) and long-term (≥12 months) mortality. The prevalence of current or previous cancer in the patients undergoing TAVR was 19.8 % (38,695 patients). Patients with cancer had a lower risk of short-term mortality (odds ratio [OR] 0.69, 95 % confidence interval [CI] 0.61–0.77, P < 0.001) but a higher risk of long-term mortality (OR 1.54, 95 % CI 1.35–1.76, P < 0.001) than those without cancer. Patients with cancer had a lower incidence of postprocedural stroke and acute kidney injury but a higher incidence of pacemaker implantation than patients without cancer.

**Conclusions:**

Patients with cancer undergoing TAVR have a good short-term prognosis and acceptable perioperative complications compared with patients without cancer. However, the long-term outcomes are contingent on cancer survival.

## 1 Introduction

Most deaths are attributable to cancer and cardiovascular disease, especially in industrialized countries, making cardio-oncology an emerging field of study [1]. Aortic stenosis (AS) is a valvular heart disease whose prevalence increases with age [2]. Because significant advancements in cancer treatment have greatly improved survival rates, there is a growing population of patients with both AS and concurrent cancer [3], [4]. The concurrent diagnosis of these two conditions in a single patient is common [5] and often presents challenging treatment decisions. Treatment of the underlying cardiovascular disease may lead to cancer-related complications, such as bleeding, whereas cancer treatment can potentially give rise to cardiac complications and exacerbate cardiovascular outcomes [6].

Current guidelines recommend transcatheter aortic valve replacement (TAVR) as the preferred treatment for patients with AS who have moderate or high surgical risk because its outcomes are comparable to those of surgical AVR [7]. Given that patients with malignancies are often deemed high-risk candidates for major cardiac surgery, TAVR has the potential to provide significant benefits to such patients [8]. However, research on the clinical efficacy and safety of TAVR in patients with cancer and severe AS is limited, and the conclusions remain controversial. Therefore, we conducted a meta-analysis to investigate the clinical outcomes in patients with previous and current cancers undergoing TAVR.

## 2 Methods

This study was performed in accordance with the Cochrane Handbook for Systematic Reviews guidelines and was reported according to the Preferred Reporting Items for Systematic Reviews and Meta-analyses (PRISMA) (Supplementary Tables 1 and 2) [9]. Ethical approval was not required for the current study, as it involved the retrieval and synthesis of data from previously published studies.

Details regarding the search strategy, inclusion and exclusion criteria, and the processes of study selection and data extraction are reported in the Supplementary Methods.

### 2.1 Outcomes definition

The primary outcomes were short-term (in-hospital or 30-day) and long-term (≥12 months) all-cause mortality. Secondary outcomes were the incidences of stroke, pacemaker implantation, acute kidney injury, major bleeding, vascular complications, and myocardial infarction.

### 2.2 Statistical analysis

Continuous data are expressed as mean ± standard deviation and categorical data are presented as absolute numbers and percentages. All analyses were conducted using Stata 17.0 statistical software (Stata Corp., College Station, TX, USA) and Easy R software based on R and R commanders (Jichi Medical University, Saitama, Japan) [10]. Meta-analyses were performed using a random-effects model. Heterogeneity was evaluated using I^2^ statistics and P-values (low, 25–50%; moderate, 50–75%; and high, >75%). Forest plots were constructed using the software. Publication bias was evaluated using a funnel plot and Egger’s test with a statistical cutoff of P = 0.1. Statistical significance was set at P < 0.05 for all other models.

## 3 Results

### 3.1 Search results and characteristics of the included studies

**Fig. 1:**
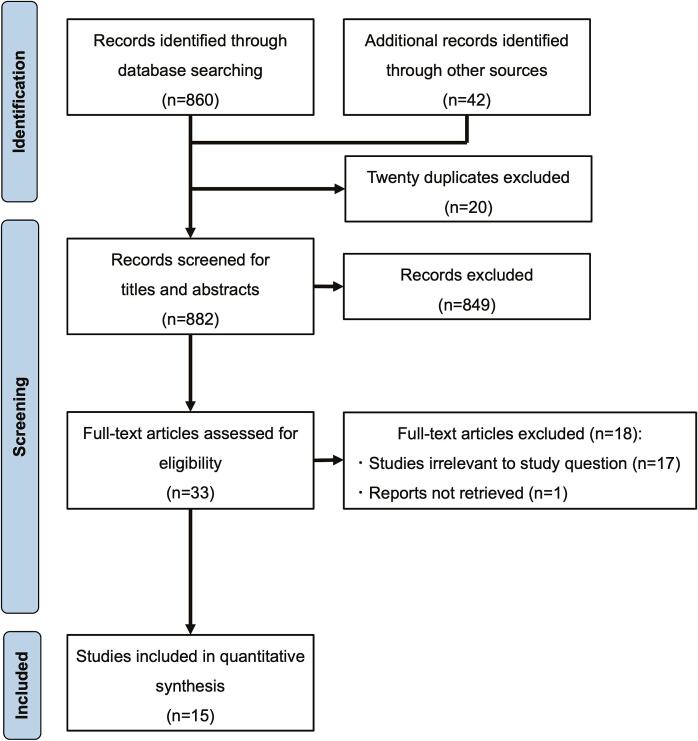
PRISMA flowchart of the study selection process. PRISMA, Preferred Reporting Items for Systematic Reviews and Meta-Analyses.

Fig. 1 shows the flow diagram of the data search and study selection process. Initially, 902 studies were identified from PubMed and Cochrane Library databases, 20 of which were duplicates. The remaining 882 studies were screened, and 849 were excluded because they were review articles, animal or cellular models, or irrelevant to this analysis. The remaining 33 studies underwent a full-text review, and 18 were excluded because they were irrelevant to the study question (n = 17) or because the full text was not retrieved (n = 1). Finally, 15 observational studies involving 195,658 patients were included, of which 38,695 (19.8%) included patients with cancer [3], [11], [12], [13], [14], [15], [16], [17], [18], [19], [20], [21], [22], [23], [24]. The baseline clinical characteristics of the patients are shown in Table 1.

**Table 1:** Characteristics of the studies included in this meta-analysis.

**Study**	**No. of patients**	**Patients with cancer (%)**	**Most common type of cancer**	**Cancer metastasis**	**Study design**	**Follow-up duration**
Kojima et al., 2022 [11]	1,114	62 (6 %)	Breast/colon/gastric	17 (27 %)	Retrospective cohort	19 months
Grant et al., 2021 [12]	123,070	23,670 (19 %)	N/A	N/A	Retrospective cohort	Hospital admission days
Karaduman et al., 2021 [13]	550	36 (7 %)	Colorectal	N/A	Retrospective cohort	30 days
Biancari et al., 2020 [14]	2,130	417 (20 %)	Breast	N/A	Retrospective cohort	7 years
Ghotra et al., 2020 [15]	1,081	181 (17 %)	Lung	N/A	Retrospective cohort	1 year
Guha et al., 2020 [16]	47,295	10,670 (23 %)	N/A	N/A	Retrospective cohort	Hospital admission days
Lantelme et al., 2020 [17]	10,221	2,050 (20 %)	N/A	N/A	Retrospective cohort	2.09 ± 1.36 years
Lind et al., 2020 [18]	1,088	249 (23 %)	Breast	16 (6 %)	Prospective cohort	10 years
Tabata et al., 2020 [19]	1,568	298 (19 %)	Prostate	68 (23 %)	Prospective cohort	5 years
Landes et al., 2019 [20]	2,744	222 (8 %)	Gastrointestinal	NA	Ambispective cohort	1 year
Tabata et al., 2019 [21]	1,204	240 (20 %)	N/A	NA	Retrospective cohort	3 years
Berkovitch et al., 2018 [22]	477	91 (19 %)	Solid tumor	NA	Retrospective cohort	851 ± 629 days
Mangner et al., 2018 [3]	1,814	349 (19 %)	Prostate	NA	Prospective cohort	3 years
van Kesteren et al., 2017 [23]	553	113 (20 %)	Lung	1 (0.1 %)	Retrospective cohort	5 years
Watanabe et al., 2016 [24]	749	47 (6 %)	Lung	N/A	Prospective cohort	272 (143–402) days

N/A, not available.

### 3.2 Clinical characteristics of the patients

Supplementary Figs. 1–7 show the differences in clinical characteristics between patients with and without cancer who underwent TAVR. Patients with cancer were more likely to be male, have a lower body mass index (BMI), and less likely to have diabetes than those without cancer (Supplementary Figs. 2–4). Age, hypertension, atrial fibrillation, and a history of myocardial infarction did not differ significantly between patients with and without cancer (Supplementary Figures 1 and 4). The prevalence of New York Heart Association (NYHA) functional class III or IV was higher in patients without cancer, the left ventricular ejection function was similar between the two groups, and the Society of Thoracic Surgeons (STS) score was significantly lower in patients with cancer than in those without cancer (Supplementary Figures 5–7). Aortic valve area was significantly smaller in the patients without cancer than those with cancer (Supplementary Figure 8).

### 3.3 Outcomes

**Fig. 2:**
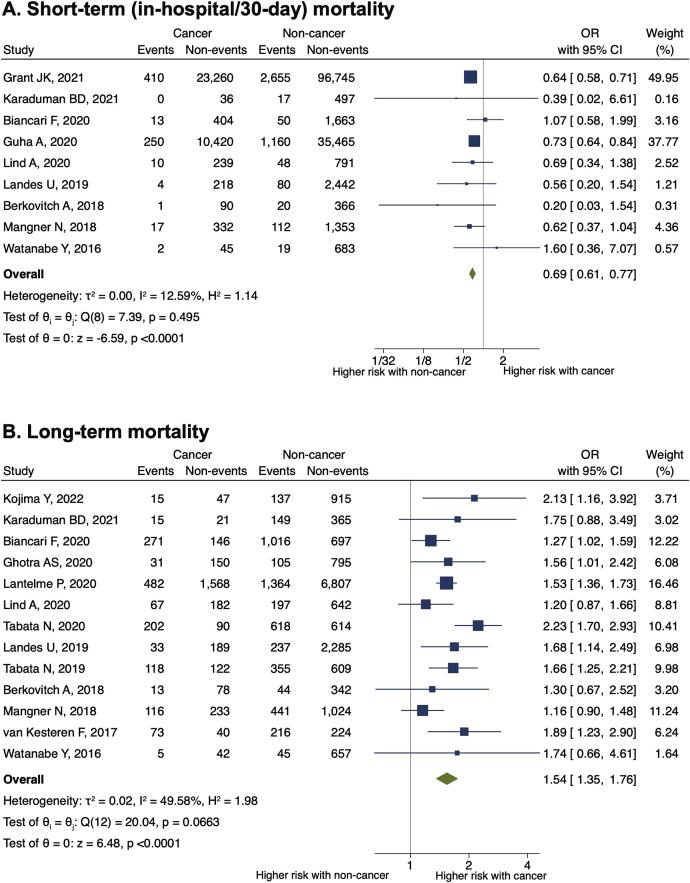
Impact of cancer on long- and short-term mortalities in patients undergoing TAVR. Forest plots showing the impact of cancer on (A) short-term and (B) long-term mortality. CI, confidence interval; OR, odds ratio; TAVR, transcatheter aortic valve replacement.

Patients with cancer had a lower risk of in-hospital or 30-day mortality (odds ratio [OR] 0.69, 95% confidence interval [CI] 0.61–0.77, P < 0.001) but a higher risk of long-term mortality (OR 1.54, 95% CI 1.35–1.76, P < 0.001) than those without cancer (Fig. 2A and 2B). We did not observe significant publication bias for short- or long-term mortality (Supplementary Figure 8). The details of causes of long-term mortality are summarized in Supplementary Table 3. The proportion of cardiovascular death as a cause of long-term mortality was equal to or lower in patients with cancer than in those without cancer. Conversely, the proportion of deaths from cancer or non-cardiovascular causes was higher in patients with cancer than in those without.

**Fig. 3:**
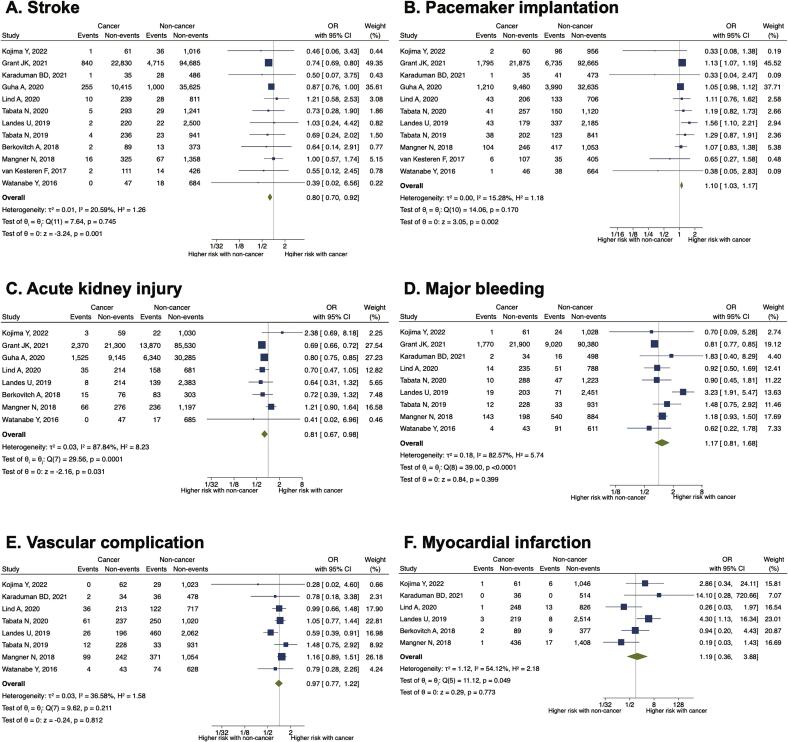
Impact of cancer on postprocedural complications. Forest plots showing the effect of cancer on (A) stroke, (B) pacemaker implantation, (C) acute kidney injury, (D) major bleeding, (E) vascular complications, and (F) myocardial infarction. CI, confidence interval; OR, odds ratio.

Patients with cancer had a lower incidence of postprocedural stroke (OR 0.80, 95% CI 0.70–0.92, P = 0.001) and acute kidney injury (AKI) (OR 0.81, 95% CI 0.67–0.98, P = 0.031) but a higher incidence of permanent pacemaker implantation (OR 1.10, 95% CI 1.03–1.17, P = 0.002) than patients without cancer (Fig. 3A-C). Post-procedural major bleeding, vascular access complications, and myocardial infarction were comparable between patients with and without cancer (Fig. 3D-3F). The incidence of paravalvular leakage after TAVR was significantly higher in patients without cancer (Supplementary Figures 9).

## 4 Discussion

### 4.1 Short-term mortality after TAVR

A key finding of this meta-analysis was that a history of cancer, including active cancer, was not associated with an overall increase in short-term mortality. The better short-term prognosis of patients with cancer may be due to a lower perioperative risk; patients with cancer had a lower BMI, incidence of diabetes, prevalence of NYHA class III and IV, and STS scores. In the 2020 ACC/AHA guideline, TAVR is not recommended in patients with a life expectancy of <1 year, even with a successful procedure, or those with a chance of “survival with benefit” of <25% at 2 years [7]. It is possible that only patients with cancer who had a good general condition were selected for TAVR, and those with a poor prognosis, such as those with terminal cancer expected to survive for less than 1 year, were excluded. Therefore, in this study, we assumed that the perioperative risk was lower in patients with cancer.

### 4.2 Perioperative events following TAVR

The lower incidence of stroke among patients with cancer may be attributed to the lower prevalence of cardiovascular disease risk factors, such as hypertension and diabetes. Previous studies have shown that the predictors of AKI risk include increased BMI, history of diabetes, hypertension, NYHA class IV, and blood transfusion [25], [26]. In this study, the higher incidence of AKI in patients without cancer may have been influenced by a higher BMI and prevalence of diabetes and hypertension.

The incidence of pacemaker implantation is significantly higher in patients with cancer. The risk factors for pacemaker implantation after TAVR have been reported to include a preexisting right bundle branch block, a self-expandable valve, and a short membranous septum [27], [28], [29]. However, in this study, due to the lack of data on electrocardiograms, valve type, and anatomical data from computed tomography imaging, the factors contributing to the increased incidence of pacemaker implantation were unknown. One possibility is that antitumor drugs and radiation therapy damage the myocardium; however, further research is required to elucidate this issue.

### 4.3 Cardiotoxic cancer therapy in patients with severe AS

Cancer therapy-related cardiac dysfunction and heart failure are well-recognized complications that negatively affect the survival and quality of life of cancer patients. Anthracyclines are the most commonly used chemotherapeutic agents for treating hematological malignancies and solid tumors. However, its efficacy is compromised by cardiotoxicity [30], [31]. Patients with preexisting severe valvular heart disease are at a high risk of anthracycline-induced cardiotoxicity, and cardio-oncology referral is recommended before anticancer therapy [32]. Discussing the risk–benefit balance of cardiotoxic anticancer treatments in high-risk patients using a multidisciplinary team (MDT) approach is also recommended [32]. Cardio-oncology services play an important role in ensuring that patients with cancer receive the best possible and safest cancer treatment, and that cardiovascular toxicity associated with cancer treatment is minimized throughout cancer treatment [33], [34].

More complex decision making may be required when asymptomatic patients with severe AS require cardiotoxic cancer therapy. Current clinical guidelines recommend AVR in patients with symptomatic severe AS, asymptomatic very severe AS, or severe AS with reduced ejection fraction or markedly elevated natriuretic peptide levels [7], [35]. However, even if asymptomatic, AS increases the left ventricular systolic pressure, leading to left ventricular dysfunction, increased myocardial oxygen consumption, and myocardial ischemia [36]. Therefore, the administration of potentially cardiotoxic drugs to such patients may induce overt heart failure symptoms and optimal cancer therapy may not be achieved. Recent clinical trials in patients without cancer suggested that early AVR may improve the prognosis of asymptomatic patients with severe AS [37]. These findings are thought-provoking, particularly in the context of the broad availability of TAVR for the treatment of AS in cancer patients who require potentially cardiotoxic cancer treatments. The decision for such patients must be made by an MDT, considering the type and stage of cancer, impact of anticancer drugs on cardiac function, urgency of cancer treatment, and availability of alternative therapies.

## 5 Limitations

This study had several limitations. First, it was conducted based on observational studies; hence, the possibility of confounding bias cannot be ignored. Second, the cause of death in these patients remains unclear. Despite the favorable short-term prognosis of patients with cancer, long-term outcomes are poor. In patients with cancer, death is suspected to be due to malignancy; however, this has not been confirmed in detail. Third, long-term prognosis after TAVR, such as after 10 years, remains unknown. Fourth, the factors contributing to the increased pacemaker implantation are unclear. Fourth, no conclusions can be drawn about the differential incidence of pacemaker implantation between patients with and without cancer owing to the lack of relevant data (e.g., ECG, CT findings, and type of transcatheter heart valve). Fifth, similarly, we also cannot draw any conclusions about the difference in PVL incidence between patients with and without cancer. This was because it was not possible to obtain data that might have influenced the incidence of paravalvular leakage, such as suboptimal prosthesis placement, incomplete apposition of the valve stent frame, or undersizing of the prosthesis. Sixth, the present study lacked information on the types, stages, and treatments of malignancy, and these cancer characteristics might have affected our results. Last, lower incidence of stroke and AKI in the cancer group cannot be adequately supported due to the missing baseline data (e.g. previous cardiac surgery, renal function, peripheral artery disease etc.) Caution should be exercised in interpreting these results.

## 6 Conclusions

Patients with cancer undergoing TAVR have a good short-term prognosis and acceptable perioperative complications compared with those without cancer. However, the long-term outcomes are contingent on cancer survival. TAVR is not only a viable option for patients with cancer but also constitutes an alternative palliative therapy. Prospective multicenter studies are necessary to further evaluate this complicated constellation.

## Funding

This work was funded by JSPS KAKENHI (23K07518) and the National Cancer Center Research and Development Fund (2023-A-12).

## CRediT authorship contribution statement

**Takumi Osawa:** Writing – original draft, Investigation, Data curation. **Kazuko Tajiri:** Project administration, Methodology, Funding acquisition, Conceptualization, Writing – review & editing. **Tomoya Hoshi:** Conceptualization, Supervision, Writing – review & editing. **Masaki Ieda:** Supervision, Writing – review & editing. **Tomoko Ishizu:** Supervision, Writing – review & editing.

## Declaration of competing interest

The authors declare that they have no known competing financial interests or personal relationships that could have appeared to influence the work reported in this paper.
