# Supplementary material for: Impact of cancer in patients with aortic stenosis undergoing transcatheter aortic valve replacement: A systematic review and meta-analysis
Source: Int J Cardiol Heart Vasc. 2024 Apr 16;52:101410. doi: 10.1016/j.ijcha.2024.101410 (PMC11033174; doi:10.1016/j.ijcha.2024.101410)
Supplement: Supplementary Data 1 [file mmc1.docx]

**Supplementay Materials**

**Supplementary Methods**

**Supplementary Table 1.** **PRISMA 2020 for abstracts checklist**

**Supplementary Table 2. PRISMA 2020 checklist**

**Supplementary Table 3. Details of causes of death during long-term follow-up**

**Supplementary Figure 1. Forest plot showing the patients’ age in cancer and non-cancer groups**

**Supplementary Figure 2. Forest plot showing the number of females in cancer and non-cancer groups**

**Supplementary Figure 3. Forest plot showing body mass index in cancer and non-cancer groups**

**Supplementary Figure 4. Forest plots showing comorbidities and past medical histories in cancer and non-cancer groups.**

**Supplementary Figure 5. Forest plot for NYHA class Ⅲ and Ⅳ in cancer and non-cancer groups**

**Supplementary Figure 6. Forest plot showing left ventricular ejection fraction in cancer and non-cancer groups**

**Supplementary Figure 7. Forest plot showing the Society of Thoracic Surgeons (STS) score in cancer and non-cancer groups**

**Supplementary Figure 8. Forest plot showing aortic valve area in cancer and non-cancer groups**

**Supplementary Figure 9. Impact of cancer on paravalvular leak**

**Supplementary Figure 10. Funnel plot showing publication bias of the included studies.**

**Supplementary Methods**

***Study search and data sources***

A search was conducted for published studies indexed in the PubMed and Cochrane Library databases to investigate all relevant articles related to TAVR and cancer published between January 2002 and January 2023. The search was initiated in 2002 when TAVR was first performed in humans.^5^ Our search strategy was structured as follows: (“cancer” OR “carcinoma” OR “sarcoma” OR “melanoma” OR “cysts” OR “cyst” OR “neoplasm s” OR “neoplasms” OR “neoplasm” OR “neurofibroma” OR “neurofibromas” OR “tumor s” OR “tumoral” OR “tumorous” OR “tumour” OR “tumor” OR “tumours” OR “tumoural” OR “tumourous” OR “tumors” OR “malignancy” OR “oncology” OR “adenocarcinoma”) AND (“transcatheter aortic valve replacement” OR “transcatheter aortic valve implantation” OR “TAVR” OR “TAVI” OR “heart valve prosthesis”).

***Selection criteria, data collection, and quality assessment***

According to our primary aim, the main inclusion criteria were studies reporting TAVR outcomes in patients with cancer, irrespective of the study design. The exclusion criteria were as follows: (a) duplicate studies, (b) studies without usable data, (c) review articles, (d) case reports, (e) case series, (f) letters, (g) animal studies, and (h) non-English studies. Two independent investigators (T.O. and K.T.) screened all the identified records (titles and abstracts) and assessed the selected full-text papers for eligibility.

**Supplementary Table 1: PRISMA 2020 for abstracts checklist**

| **Section and Topic** | **Item #** | **Checklist item** | **Reported (Yes/No)** |
| --- | --- | --- | --- |
| **TITLE** |  |  |  |
| **Title** | 1 | Identify the report as a systematic review. | Yes |
| **BACKGROUND** |  |  |  |
| **Objectives** | 2 | Provide an explicit statement of the main objective(s) or question(s) the review addresses. | Yes |
| **METHODS** |  |  |  |
| **Eligibility criteria** | 3 | Specify the inclusion and exclusion criteria for the review. | No |
| **Information sources** | 4 | Specify the information sources (e.g., databases, registers) used to identify studies and the date when each was last searched. | Yes |
| **Risk of bias** | 5 | Specify the methods used to assess risk of bias in the included studies. | No |
| **Synthesis of results** | 6 | Specify the methods used to present and synthesise results. | No |
| **RESULTS** |  |  |  |
| **Included studies** | 7 | Give the total number of included studies and participants and summarise relevant characteristics of studies. | Yes |
| **Synthesis of results** | 8 | Present results for main outcomes, preferably indicating the number of included studies and participants for each. If meta-analysis was done, report the summary estimate and confidence/credible interval. If comparing groups, indicate the direction of the effect (i.e. which group is favoured). | Yes |
| **DISCUSSION** |  |  |  |
| **Limitations of evidence** | 9 | Provide a brief summary of the limitations of the evidence included in the review (e.g. study risk of bias, inconsistency and imprecision). | Yes |
| **Interpretation** | 10 | Provide a general interpretation of the results and important implications. | Yes |
| **OTHER** |  |  |  |
| **Funding** | 11 | Specify the primary source of funding for the review. | No |
| **Registration** | 12 | Provide the register name and registration number. | No |

**Supplementary Table 2: PRISMA 2020 checklist**

| **Topic** | **Item #** | **Item** | **Page** |
| --- | --- | --- | --- |
| **TITLE** |  |  |  |
| **Title** | 1 | Identify the report as a systematic review. | 1 |
| **ABSTRACT** |  |  |  |
| **Abstract** | 2 | See the PRISMA 2020 for abstracts checklist | 3 |
| **INTRODUCTION** |  |  |  |
| **Rationale** | 3 | Describe the rationale for the review in the context of existing knowledge. | 4 |
| **Objectives** | 4 | Provide an explicit statement of the objective(s) or question(s) the review addresses. | 4 |
| **METHODS** |  |  |  |
| **Eligibility criteria** | 5 | Specify the inclusion and exclusion criteria for the review and how studies were grouped for the syntheses. | 5 |
| **Information sources** | 6 | Specify all databases, registers, websites, organisations, reference lists and other sources searched or consulted to identify studies. Specify the date when each source was last searched or consulted. | 5 |
| **Search strategy** | 7 | Present the full search strategies for all databases, registers and websites, including any filters and limits used. | 5 |
| **Selection process** | 8 | Specify the methods used to decide whether a study met the inclusion criteria of the review, including how many reviewers screened each record and each report retrieved, whether they worked independently, and if applicable, details of automation tools used in the process. | 5-6 |
| **Data collection process** | 9 | Specify the methods used to collect data from reports, including how many reviewers collected data from each report, whether they worked independently, any processes for obtaining or confirming data from study investigators, and if applicable, details of automation tools used in the process. | 5-6 |
| **Data items** | 10a | List and define all outcomes for which data were sought. Specify whether all results that were compatible with each outcome domain in each study were sought (e.g. for all measures, time points, analyses), and if not, the methods used to decide which results to collect. | 6 |
|  | 10b | List and define all other variables for which data were sought (e.g. participant and intervention characteristics, funding sources). Describe any assumptions made about any missing or unclear information. | 6 |
| **Study risk of bias assessment** | 11 | Specify the methods used to assess risk of bias in the included studies, including details of the tool(s) used, how many reviewers assessed each study and whether they worked independently, and if applicable, details of automation tools used in the process. | 6 |
| **Effect measures** | 12 | Specify for each outcome the effect measure(s) (e.g. risk ratio, mean difference) used in the synthesis or presentation of results. | 6 |
| **Synthesis methods** | 13a | Describe the processes used to decide which studies were eligible for each synthesis (e.g. tabulating the study intervention characteristics and comparing against the planned groups for each synthesis (item 5)). | 6 |
|  | 13b | Describe any methods required to prepare the data for presentation or synthesis, such as handling of missing summary statistics, or data conversions. | Not Applicable |
|  | 13c | Describe any methods used to tabulate or visually display results of individual studies and syntheses. | 6 |
|  | 13d | Describe any methods used to synthesize results and provide a rationale for the choice(s). If meta-analysis was performed, describe the model(s), method(s) to identify the presence and extent of statistical heterogeneity, and software package(s) used. | 6 |
|  | 13e | Describe any methods used to explore possible causes of heterogeneity among study results (e.g. subgroup analysis, meta-regression). | Not Applicable |
|  | 13f | Describe any sensitivity analyses conducted to assess robustness of the synthesized results. | Not Applicable |
| **Reporting bias assessment** | 14 | Describe any methods used to assess risk of bias due to missing results in a synthesis (arising from reporting biases). | 6 |
| **Certainty assessment** | 15 | Describe any methods used to assess certainty (or confidence) in the body of evidence for an outcome. | Not Applicable |
| **RESULTS** |  |  |  |
| **Study selection** | 16a | Describe the results of the search and selection process, from the number of records identified in the search to the number of studies included in the review, ideally using a flow diagram. | 6-7 |
|  | 16b | Cite studies that might appear to meet the inclusion criteria, but which were excluded, and explain why they were excluded. | 6-7 |
| **Study characteristics** | 17 | Cite each included study and present its characteristics. | 7 |
| **Risk of bias in studies** | 18 | Present assessments of risk of bias for each included study. | 8  Supplimentary Figure 8 |
| **Results of individual studies** | 19 | For all outcomes, present, for each study: (a) summary statistics for each group (where appropriate) and (b) an effect estimate and its precision (e.g. confidence/credible interval), ideally using structured tables or plots. | 8 |
| **Results of syntheses** | 20a | For each synthesis, briefly summarise the characteristics and risk of bias among contributing studies. | 7-8 |
|  | 20b | Present results of all statistical syntheses conducted. If meta-analysis was done, present for each the summary estimate and its precision (e.g. confidence/credible interval) and measures of statistical heterogeneity. If comparing groups, describe the direction of the effect. | 7-8  Supplimentary Fig. 1-7 |
|  | 20c | Present results of all investigations of possible causes of heterogeneity among study results. | Not Applicable |
|  | 20d | Present results of all sensitivity analyses conducted to assess the robustness of the synthesized results. | Not Applicable |
| **Reporting biases** | 21 | Present assessments of risk of bias due to missing results (arising from reporting biases) for each synthesis assessed. | Not Applicable |
| **Certainty of evidence** | 22 | Present assessments of certainty (or confidence) in the body of evidence for each outcome assessed. | Not Applicable |
| **DISCUSSION** |  |  |  |
| **Discussion** | 23a | Provide a general interpretation of the results in the context of other evidence. | 8 |
|  | 23b | Discuss any limitations of the evidence included in the review. | 12 |
|  | 23c | Discuss any limitations of the review processes used. | 12 |
|  | 23d | Discuss implications of the results for practice, policy, and future research. | 8-12 |
| **OTHER INFORMATION** |  |  |  |
| **Registration and protocol** | 24a | Provide registration information for the review, including register name and registration number, or state that the review was not registered. | Not Applicable |
|  | 24b | Indicate where the review protocol can be accessed, or state that a protocol was not prepared. | Not Applicable |
|  | 24c | Describe and explain any amendments to information provided at registration or in the protocol. | Not Applicable |
| **Support** | 25 | Describe sources of financial or non-financial support for the review, and the role of the funders or sponsors in the review. | 2 |
| **Competing interests** | 26 | Declare any competing interests of review authors. | 2 |
| **Availability of data, code and other materials** | 27 | Report which of the following are publicly available and where they can be found: template data collection forms; data extracted from included studies; data used for all analyses; analytic code; any other materials used in the review. | 13 |

**Supplementary Table 3. Details of causes of death during long-term follow-up**

| **Study** | **Cause of death in patients with cancer** | **Cause of death in patients without cancer** |
| --- | --- | --- |
| Kojima et al., 2022^7^ | Cancer (73%) | Cancer (5.1%) |
| Karaduman et al., 2021^9^ | N/A | N/A |
| Biancari et al., 2020^10^ | CVD (69.3%)  Cancer (34.6%) | CVD (70.8%)  Cancer (18.5%) |
| Ghotra et al., 2020^11^ | N/A | N/A |
| Lantelme et al., 2020^13^ | N/A | N/A |
| Lind et al., 2020^14^ | N/A | N/A |
| Tabata et al., 2020^15^ | Of the 14 cases where the cause of death could be determined, 12 (85.7%) were non-CVD | N/A |
| Landes et al., 2019^16^ | Non-CVD (75%) | N/A |
| Tabata et al., 2019^17^ | N/A | N/A |
| Berkovitch et al., 2018^18^ | CVD (23.1%) | CVD (43.2%) |
| Mangner et al., 2018^19^ | CVD (52%)  Non-CVD (48%) | CVD (80%)  Non-CVD (20%) |
| van Kesteren et al., 2017^20^ | CVD (68%)  Non-CVD (22%)  Cancer (6.2%) | CVD (75%)  Non-CVD (15%)  Caner (3.6%) |
| Watanabe et al., 2016^21^ | CVD (0%)  Cancer (60%)  - | CVD (7%)  -  Non-CVD (88.9%) |

CVD, cardiovascular disease; N/A, not available.

**
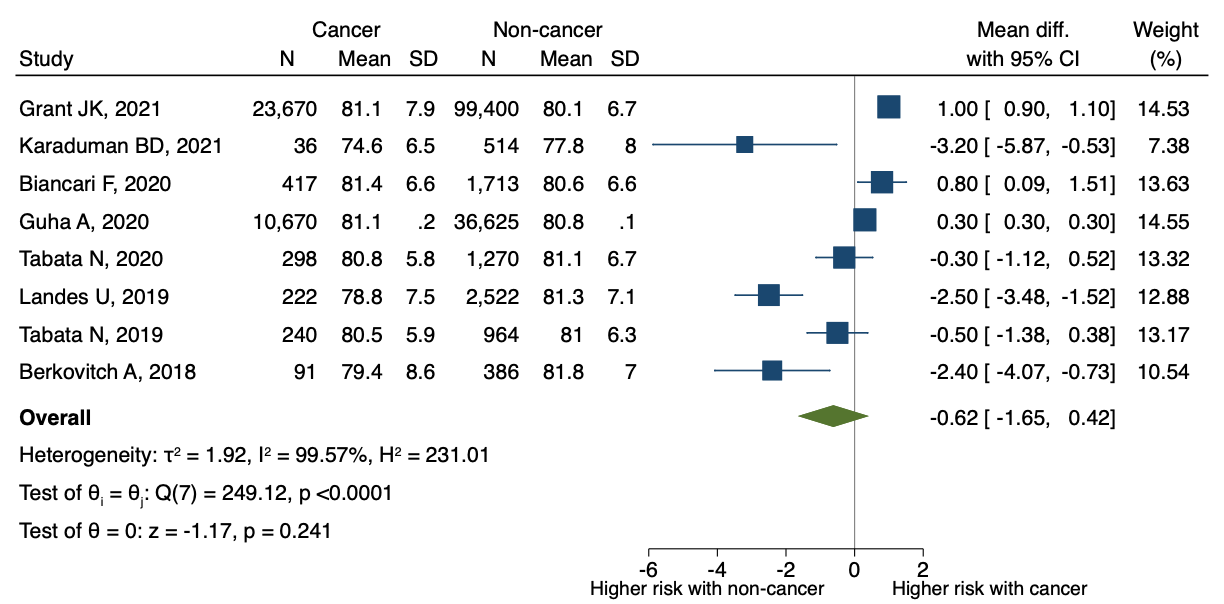
**

**Supplementary Figure 1. Forest plot showing the patients’ age in the cancer and non-cancer groups**

**
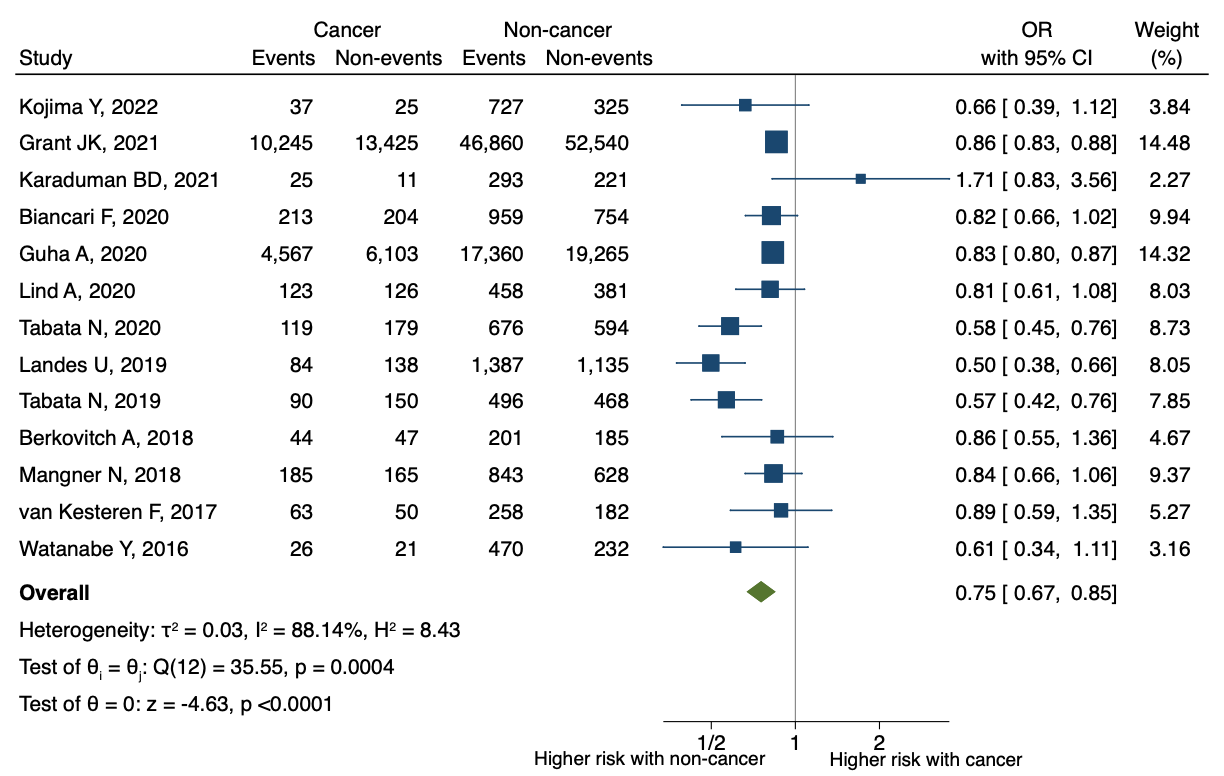
**

**Supplementary Figure 2. Forest plot showing the number of females in the cancer and non-cancer groups**

**
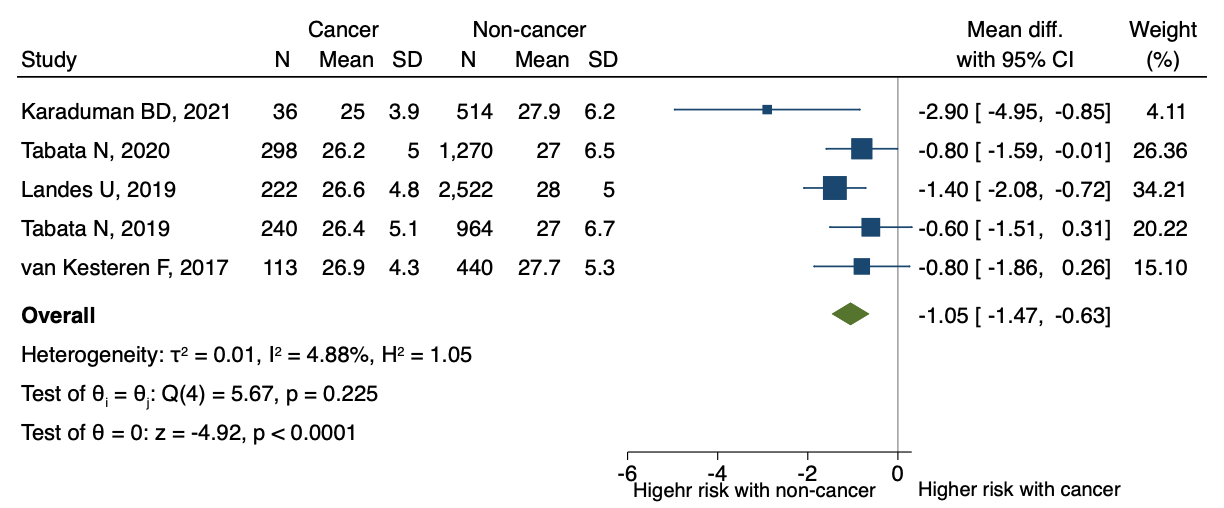
**

**Supplementary Figure 3. Forest plot showing body mass index in the cancer and non-cancer groups**

**Supplementary Figure 4. Forest plots showing comorbidities and past medical histories in cancer and non-cancer groups.** (A) hypertension, (B) diabetes, (C) atrial fibrillation, and (D) myocardial infarction.


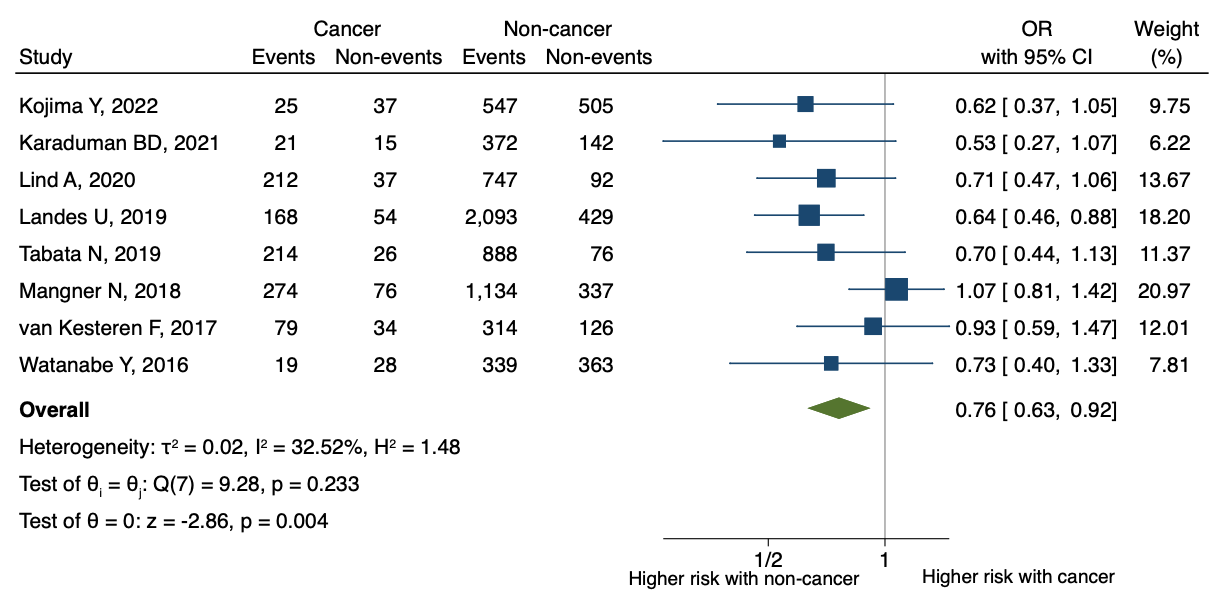


**Supplementary Figure 5. Forest plot for NYHA class Ⅲ and Ⅳ in cancer and non-cancer groups**


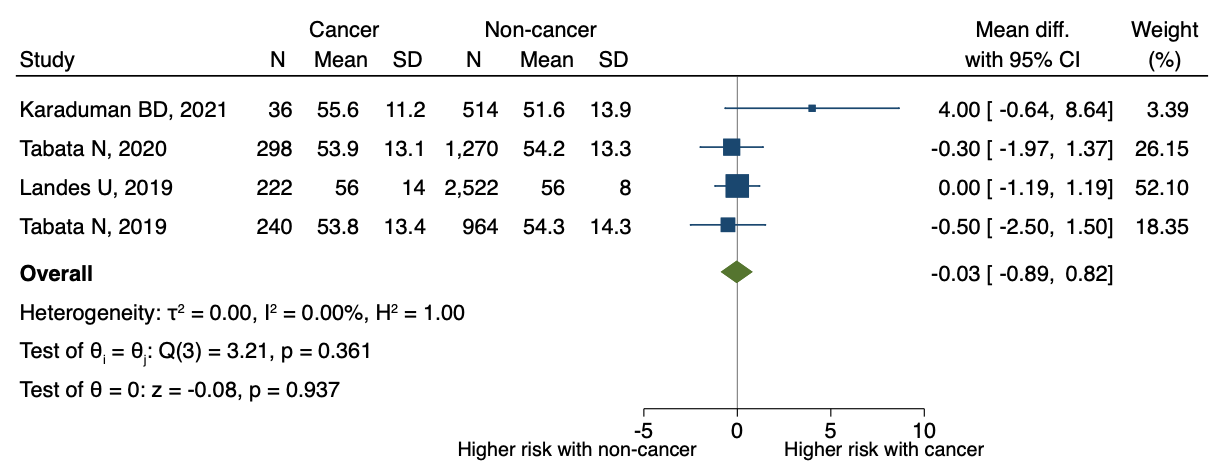


**Supplementary Figure 6. Forest plot showing left ventricular ejection fraction in cancer and non-cancer groups**


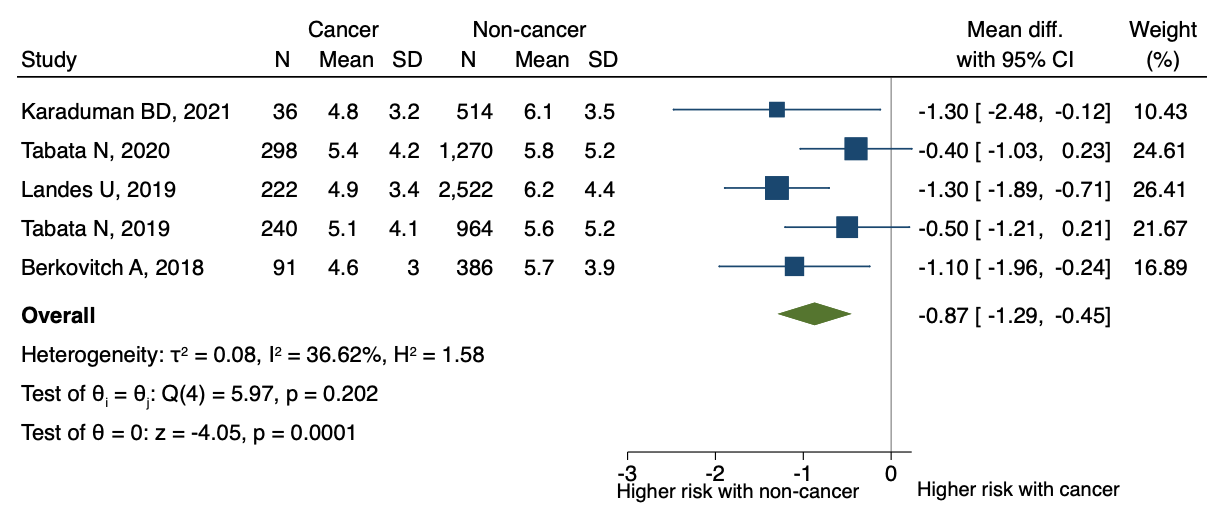


**Supplementary Figure 7. Forest plot showing the Society of Thoracic Surgeons (STS) score in cancer and non-cancer groups**

**Supplementary Figure 8. Forest plot showing aortic valve area in cancer and non-cancer groups**

**Supplementary Figure 9. Impact of cancer on paravalvular leak**

**Supplementary Figure 10. Funnel plot showing publication bias of the included studies.**

(A) Short-term mortality and (B) long-term mortality.
